# Implementation and adoption of advanced care planning in the elderly trauma patient

**DOI:** 10.1186/s13017-018-0201-6

**Published:** 2018-09-06

**Authors:** K. Verhoeff, P. Glen, A. Taheri, B. Min, B. Tsang, V. Fawcett, S. Widder

**Affiliations:** 1 Faculty of Medicine and Dentistry, University of Alberta, 8440-112 St. Edmonton, Edmonton, Alberta T6G 2B7 Canada; 2 Department of Surgery, 2D4.27 Mackenzie Health Sciences Centre, University of Alberta, 8440-112 St. Edmonton, Edmonton, Alberta T6G 2B7 Canada

**Keywords:** Quality improvement, Geriatric trauma, Advanced care planning, Patient and family-centered care

## Abstract

**Background:**

Geriatric trauma has high morbidity and mortality, often requiring extensive hospital stays and interventions. The number of geriatric trauma patients is also increasing significantly and accounts for a large proportion of trauma care. Specific geriatric trauma protocols exist to improve care for this complex patient population, who often have various comorbidities, pre-existing medications, and extensive injury within a trauma perspective. These guidelines for geriatric trauma care often suggest early advanced care planning (ACP) discussions and documentation to guide patient and family-centered care.

**Methods:**

A provincial ACP program was implemented in April of 2012, which has since been used by our level 1 trauma center. We applied a before and after study design to assess the documentation of goals of care in elderly trauma patients following implementation of the standardized provincial ACP tool on April 1, 2012.

**Results:**

Documentation of ACP in elderly major trauma patients following the implementation of this tool increased significantly from 16 to 35%. Additionally, secondary outcomes demonstrated that many more patients received goals of care documentation within 24 h of admission, and 93% of patients had goals of care documented prior to intensive care unit (ICU) admission. The number of trauma patients that were admitted to the ICU also decreased from 17 to 5%.

**Conclusion:**

Early advanced care planning is crucial for geriatric trauma patients to improve patient and family-centered care. Here, we have outlined our approach with modest improvements in goals of care documentation for our geriatric population at a level 1 trauma center. We also outline the benefits and drawbacks of this approach and identify the areas for improvement to support improved patient-centered care for the injured geriatric patient. Here, we have provided a framework for others to implement and further develop.

**Electronic supplementary material:**

The online version of this article (10.1186/s13017-018-0201-6) contains supplementary material, which is available to authorized users.

## Background

The number of Canadian seniors is increasing significantly, with people over the age of 65 expected to compose 25% of the country’s population by 2036 [1]. A proportional increase in geriatric trauma is expected [2, 3]. Geriatric trauma is associated with longer intensive care unit (ICU) admissions, more life-threatening complications while in hospital, and significantly higher mortality despite similar injury severity when compared to younger patients [2–6].

The American College of Surgeons Committee on Trauma (ACS COT) has defined specific protocols for geriatric trauma in order to improve management of comorbid conditions, polypharmacy, poor physiologic reserve, and elderly specific complications (i.e., delirium) [2]. ACS COT suggests involving the patient, surrogate decision-makers, family, and the health care team in a discussion about the patient’s treatment priorities. This conversation aims to establish and document the commonly termed “goals of care” (GOC) within the first 72 h after admission and is meant to weigh the benefits of escalating treatments against the risks of complications [2].

Despite trauma being a leading cause of death in Canada, there is a relative dearth of studies focusing on GOC discussions in trauma patients [7]. Advanced care planning (ACP) facilitates discussions around patient’s health care goals (i.e., cardiopulmonary resuscitation) along with a realistic presentation of what intensive, invasive treatments can provide. ACP processes can guide complex treatment decision-making (including end-of-life discussions between allied health professionals, patients, and their families) to promote patient and family-centered care (PFCC) and eliminate uncertainty regarding the patient’s wishes [8, 9].

The province of Alberta implemented an ACP tool in April of 2012, including within our level 1 trauma center. The program involved providing every patient and family with information sheets regarding ACP, prompting a discussion about future care between the patient and care providers regarding the patient’s care goals, and ultimately documenting these goals of care in the chart (see Additional file 1). A specific GOC order sheet was also provided to practitioners for process standardization (see Additional file 1). We sought to determine the effect of this ACP tool on establishing documented GOC for geriatric trauma patients. We also aimed to outline the benefits and drawbacks of this approach and identify the areas for improvement to assist others who wish to implement an ACP system within their trauma systems.

## Methods

We applied a before and after study design to assess the documentation of GOC in elderly trauma patients following implementation of the standardized provincial ACP tool on April 1, 2012 (see Additional file 1). The Alberta Trauma Registry is a prospective database that collects information on all major trauma patients (Injury Severity Score ≥ 12). This database was retrospectively queried for a cohort of patients over the age of 65 who were admitted from April 1, 2010, to September 30, 2014, representing a period of 24 months before and 30 months after ACP implementation. Charts for this cohort were individually reviewed to determine patient demographics, whether GOC were documented, whether it was done within 24 h of admission and prior to ICU admission, the source of the GOC information, and the patient’s post-hospitalization disposition.

Data was separated into a pre-intervention group (*n* = 177) that included patients 24 months prior to April 2012 and a post-intervention group (*n* = 294) that included patients 30 months after ACP program implementation. A 2-year before and after time frame was chosen to adequately assess for change following the project implementation. As the ACP tool was implemented provincially, an additional 6 months was included within the analysis for the after group to allow for a period of institutional uptake of these processes. The groups were compared based on Injury Severity Score (ISS), sex, age, length of stay, and mechanism of injury, using chi-squared tests of independence. The primary outcome measure of this study was whether the ACP intervention was associated with a change in the documentation of GOC in the chart overall; a comparison between both time periods was made using Fisher’s exact test. Secondary measures assessed documentation of GOC within 24 h and upon admission to ICU. These were also assessed with Fisher’s exact test.

## Results

The two groups of geriatric trauma patients in this study were similar with regard to age, sex, mechanism of injury, length of stay, ISS, and disposition after discharge (Table 1). Patients were approximately 77 years old in both groups with 35% of patients before 2012 and 40% after 2012 being female (Table 1). Importantly, the mean ISS for patients in both groups was statistically similar, being 23 before the ACS program and 22 after. Patients primarily suffered blunt traumatic injuries from either falls or motor vehicle accidents, a distribution that did not change between study periods (Table 1).

**Table 1:** Patient demographics

	Before (*n* = 177)	After (*n* = 294)	p value
Mean age (years)	76.50	77.02	0.51
Mean ISS	22.65	22.13	0.50
Median LOS (days)	9	7	N/A
Number female (proportion)	60 (0.34)	117 (0.40)	0.92
Number male (proportion)	117 (0.66)	177 (0.60)	0.94
Injury	*n* (%)	*n* (%)	
Blunt	174 (98)	289 (98)	0.99
Fall	118 (68)	207 (72)	0.96
Motor vehicle	35 (20)	57 (20)	0.99
Pedestrian	8 (5)	9 (3)	0.94
Other	13 (7)	16 (6)	0.94
Penetrating	0 (0)	1 (0)	0.94
Burn	3 (2)	4 (1)	0.98
Other	0 (0)	1 (0)	0.94
Disposition			
Home	73 (41)	127 (43)	0.97
Home support	13 (7)	10 (3)	0.84
Other hospital	35 (20)	49 (17)	0.94
Rehabilitation	13 (7)	30 (10)	0.92
Chronic care	20 (11)	27 (9)	0.95
Nursing home	7 (4)	4 (1)	0.83
Died	16 (9)	47 (16)	0.84

Characteristics of geriatric trauma population before and after the implementation of the advanced care planning project at our hospital

Analysis of the primary outcome demonstrated a statistically significant change (*p* < 0.01) in GOC documentation following the ACP program implementation. Twenty-four months prior to the ACP program implementation, only 16% of major geriatric trauma patients had documented GOC by the end of their hospital stay (Table 2). This improved to 35% in the study period following ACP project implementation.

**Table 2:** Successful goals of care documentation

Goals of care	Before (*n* = 177)	After (*n* = 294)	*p* value
	*n* (%)	*n* (%)	
Documented			
Yes	28 (16)	103 (35)	
No	149 (84)	191 (65)	< 0.01
Within 24 h			
Yes	20 (11)	61 (21)	
No	157 (89)	233 (79)	< 0.01
Source			
Patient	1 (4)	2 (2)	
Substitute decision-maker	26 (93)	87 (84)	
Advance directive	1 (4)	14 (14)	ns

Documentation of goals of care before and after the implementation of the advanced care planning project at our hospital

Secondary outcomes demonstrated that GOC documentation within 24 h was significantly higher after the intervention (Table 2). For the 30 months following April 2012, 21% of geriatric major trauma patients had this documentation done within 24 h as compared to 11% in the 24 months prior to ACP implementation.

The rate of admission to the ICU was significantly lower after the ACP program implementation when compared to before (Table 3). There was also a significant increase in the documentation of GOC for patients admitted to the ICU with 93% of admitted ICU patients having this documented after the ACP implementation, as compared to 20% prior to this project.

**Table 3:** Admission to ICU and rate of GOC documentation

	Before (*n* = 177)	After (*n* = 294)	*p* value
	*n* (%)	*n* (%)	
Admitted to ICU	30 (17)	14 (5)	< 0.01
Goals documented			
Yes	6 (20)	13 (93)	
No	24 (80)	1 (7)	< 0.01

Documentation of goals of care before and after the implementation of the advanced care planning project for patients admitted to the intensive care unit (ICU)

## Discussion

Despite the importance of ACP, a 2008 report from the US Congress demonstrated the widespread failure of implementation [10]. Some publications have advocated to abandon advanced care directives, citing reasons such as patient uncertainty about their use, inadequate prognostication to guide discussions, and poor compliance by health care practitioners with patient wishes [11–13]. However, when achieved ACP has been shown to significantly reduce anxiety, depression, and post-traumatic stress disorder in the relatives of patients who die [8, 14, 15]. Even when ACP was done but not enacted, patient and family satisfaction with their care was significantly improved [14]. This correlation addresses the vital importance of patient autonomy and PFCC to guide treatment decisions. It is also vitally important to separate the notion of ACP and end of life care decision-making. The two are independent, with the ACP discussion establishing patient care priorities. If the patient’s priorities do not fit with achievable improvements in their condition, then end of life care discussions may be required. However, the initial discussion should focus on establishing priorities in care. We outline our institution’s improvements for geriatric trauma patients following the implementation of a provincial ACP tool and discuss the areas for potential improvement.

As the management of traumatic injuries improves and patient expectations of autonomy are reinforced, the practice of ACP will become increasingly important [3–6]. Prior to the implementation of Alberta’s provincial ACP program, only 16% of our level one trauma center’s major trauma patients over 65 years old had documented GOC by the end of their hospital stay (Table 2). Following the implementation of a provincial ACP program, GOC documentation changed significantly, with an increase to 34% (Table 2). Although we achieved improvement, this result shows that many patients did not have their goals documented—indicating that barriers continue to exist that limit ACP discussions. The source of the GOC decision was documented in our study and shows that in both the before and after groups, most decisions regarding GOC were provided by a substitute decision-maker or an advanced care directive (Table 2). This is likely because geriatric trauma patients have injuries, co-morbidities, and complications (i.e., delirium) that would prevent them from making GOC decisions. This also highlights the importance of care conferences that focus on PFCC early during geriatric trauma admission to support GOC decisions.

Due to the high risk of intervention, prolonged ICU admission, life-threatening complications, and mortality in geriatric trauma, early ACP discussion is key [2, 4–6]. Following implementation of our ACP tool, new ACS COT guidelines suggested ACP and documentation of GOC within 72 h of admission for geriatric trauma patients [2]. Secondary outcomes to evaluate GOC documentation in the first 24 h and prior to ICU admission were chosen as two identifiable time points when geriatric patients were likely to undergo interventions. However, if ACS COT guidelines were released prior to study implementation, a 72-h period may have been chosen to assess our compliance with these guidelines. Despite that, our ACP program implementation was associated with increased early GOC documentation within the first 24 h (Table 2).

A substantial increase in GOC prior to ICU admission is also noted from 20 to 93% with an associated decrease in ICU admission from 17 to 5% (Table 3). There was an obvious culture shift within the ICU to initiate GOC documentation on transfer to their unit, which may have even prevented undesired transfers to the ICU, whereby risks of intensive invasive care would have outweighed benefits to the patient. We suggest that ICU providers are likely accustomed to undergoing ACP discussion due to their highly acute patient population and specialized training around PFCC and communication, lending to the ease of transition for their unit. It may be beneficial to involve these types of practitioners in GOC discussion early in a geriatric trauma patient’s hospital admission. Having these practitioners involved with medical education to provide tools and approaches for these discussions may also be beneficial.

Of note, there was a statistically insignificant mortality increase after ACP project implementation (Table 1). This may relate to fewer patients desiring ICU care and heroic lifesaving measures. There was also an accompanying statistically insignificant trend towards fewer patients sent to chronic care or a nursing home following their trauma admission. However, these results should be evaluated cautiously, as our study lacked the power to isolate the significance of these trends.

Successful programs such as “Respecting Choices,” “Physician Orders for Life-Sustaining Treatment,” and others outlined important tools for ACP implementation that our provincial program modeled [14, 16]. These included a need for leaders to enact change, implementation in a medical culture receptive to change, and complex ACP that focused on discussions rather than specific interventions [14, 16]. Ensuring ACP and documentation of GOC prior to deterioration in condition is vitally important to ensure escalated care can be accessed expediently, when appropriate [10, 16]. The ACP program also increased GOC documentation in the first 24 h (Table 2), which is important for geriatric trauma patients who often require rapid interventions soon after admission. A final important aspect of successful ACP project implementation that we modeled is an openness to department-specific quality improvement, as ACP is diverse and can take many forms even within a single hospital [14–16]. This will allow our project to continue building on its success in the future within our own institution as we seek opportunities to educate our physicians on the importance of having ACP discussions.

Despite ACP program implementation and a significant change in GOC documentation, we are seeking ways to increase the documentation beyond the 35% level we have achieved in our current study. Achievable, identifiable, and clinically significant pathways to implementing effective ACP have been demonstrated, and we continue to follow these models to further improve our ACP practices [8, 14–16]. The barriers to ACP in these studies include staff availability and time, their ability to confidently and accurately discuss ACP, and organizational commitment and support for ACP programs [14, 17–19]. Similarly, focusing on specific advanced directives, rather than a complex and complete understanding of the patient’s GOC, leads to non-documented and ineffective ACP [13, 14, 19, 20].

### Lessons and limitations

Future initiatives that may assist our trauma services division to improve ACP for geriatric trauma patients exist. Ensuring ACP prior to emergency care, such as by primary care providers in family practice—so-called advance directives—has been used by other programs to improve ACP and reduce time constraints for acute care providers in the emergency setting [10, 16]. It is important to recognize that while ACP was increased through this project, 93% of GOC decisions in the before group and 84% in the after group were made via a substitute decision maker; this highlights the importance of discussing ACP while patients are healthy to maintain their autonomy in acute scenarios. Initiating these family discussions and decisions should be standard of care for all patients that are admitted to hospital and should be encouraged in the outpatient population as well. Emphasizing the importance of ACP during trauma educational initiatives and outreach programs within the community may improve pre-trauma GOC documentation or discussion; as mentioned, involving experienced intensivists to assist education may provide insight into tools and approaches for these difficult discussions.

**Fig. 1:**
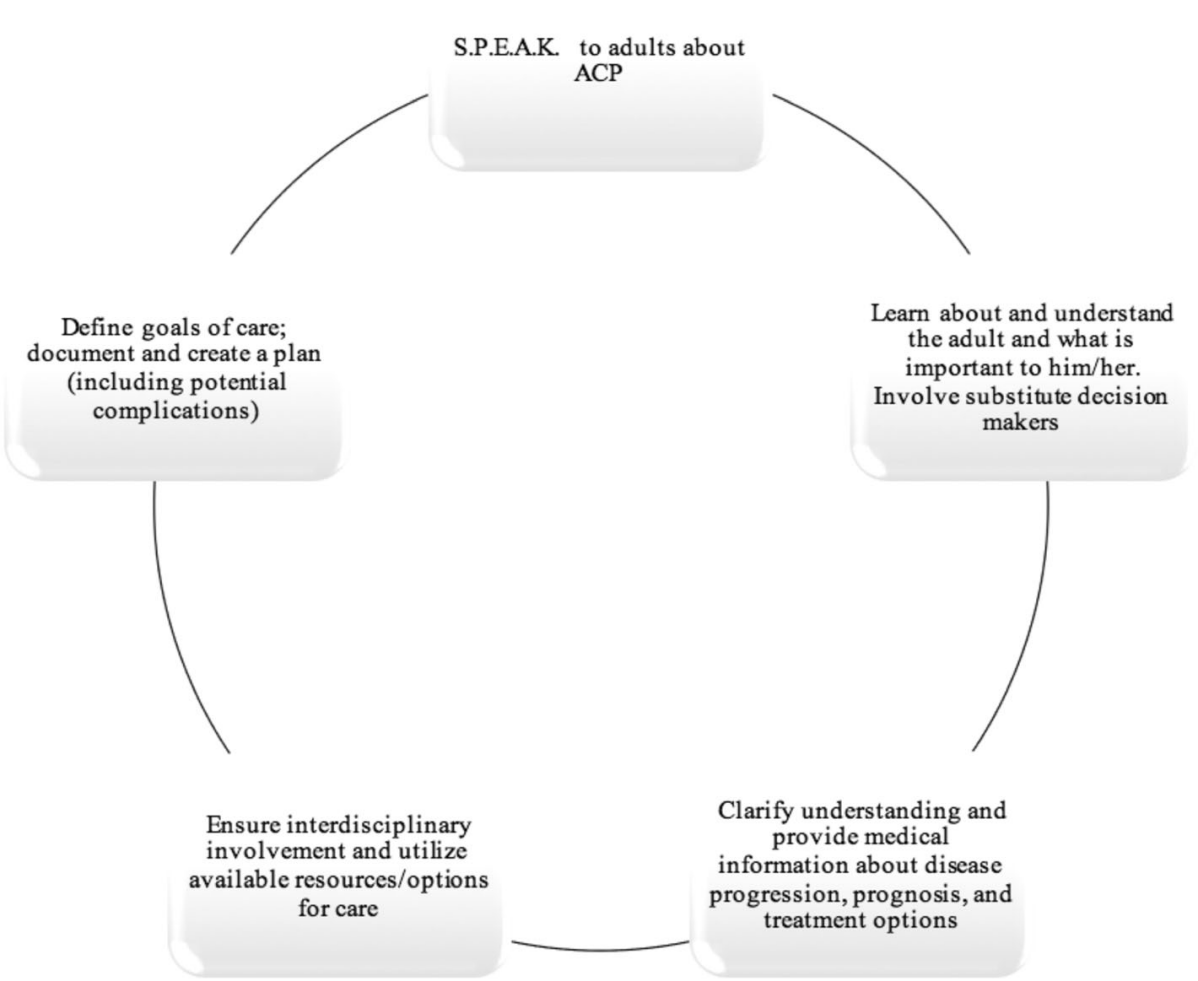
The five “Core Elements of ACP.” Five elements of advanced care planning identified by our provincial advanced care planning program to help involve allied health professionals in goals of care discussions [16]

Time constraint concerns both in the primary care and emergency care settings were identified by practitioners early in the development of model ACP programs and may be an area for future examination [16]. While the resources do not exist to employ individuals solely for the purposes of ACP discussion, the provincial program suggested that all health care practitioners (including nurses, social workers, respiratory therapists, spiritual health practitioners, and all allied health professionals) play a role in ACP by following five “Core Elements of ACP” (Fig. 1) [16]. Involving specialists, such as intensivists, geriatricians, and palliative care specialists, early in the care of geriatric trauma patients is likely beneficial, especially if a shared mental model between the care team and patient and family cannot be achieved. In order to implement these elements in our program and limit time constraint issues, the culture and approach to ACP will likely require champions for change to lead the way, as other successful programs have demonstrated [14, 16].

Despite demonstrating improved GOC documentation, the authors recognize certain shortcomings of this method for evaluating ACP program implementation. A before and after study design cannot exclude that other changes or that baseline improvement led to the results we discovered. An interrupted time series analysis to describe the baseline increase in GOC documentation could help attribute the results of our study more directly to the intervention. Also, our comparison groups would ideally be similar in size; however, this is likely accounted for by increasing geriatric trauma volume at our center and the 6-month transition period in our post-intervention group. Our study also associated GOC documentation with ACP discussions; however, we did not evaluate the comprehensiveness of the ACP discussions, or whether they led to a change in intervention and procedures for geriatric trauma patients. Additional studies would be needed to evaluate if the change in GOC documentation and ACP over the study period altered or improved care. Our study attempted to assess mortality and disposition but did not possess sufficient power to determine any change. Future studies may endeavor to include these changes and assess other clinical outcomes to better evaluate the implementation of their ACP programs.

## Conclusion

The provincial advanced care planning program applied to major geriatric trauma patients at our hospital successfully increased GOC documentation from 16 to 35%. Additionally, 21% of patients received had documentation within the first 24 h of their admission versus 11% prior to the project. This demonstrates that ACP programs can improve GOC documentation within the geriatric trauma environment. Additional quality improvement is required to further increase ACP use at our hospital; however, many other programs have shown successful ACP implementation in similar patients and provide various areas for improvement [14–16]. We posit that the most significant factors for ACP improvement using our program will involve additional educational support on the importance of these discussions, the involvement of specialists’ adept at these discussions, and leaders to promote effective culture change about ACP at our hospital. While this process can be slow, as demonstrated by others, effective ACP is required for proper patient-centered care for the growing geriatric trauma patient population.

## Additional file

Additional file 1: Advanced care planning bedside tool and order set. Pdf. file that outlines the advanced care planning “goals of care” order set and a document that is provided to patients and their families to assist with decision making and thoughtful advanced care planning. (PDF 2053 kb)

## Abbreviations

ACP: Advanced care planning
ACS COT: American College of Surgeons Committee on Trauma
GOC: Goals of care
ICU: Intensive care unit
ISS: Injury Severity Score
PFCC: Patient and family-centered care
