# Supplementary material for: Implementation and adoption of advanced care planning in the elderly trauma patient
Source: World J Emerg Surg. 2018 Sep 6;13:40. doi: 10.1186/s13017-018-0201-6 (PMC6127940; doi:10.1186/s13017-018-0201-6)
Supplement: Supplementary file 1 — Advanced care planning bedside tool and order set. Pdf. file that outlines the advanced care planning “goals of care” order set and a document that is provided to patients and their families to assist with decision making and thoughtful advanced care planning. (PDF 2053 kb) [file 13017_2018_201_MOESM1_ESM.pdf]

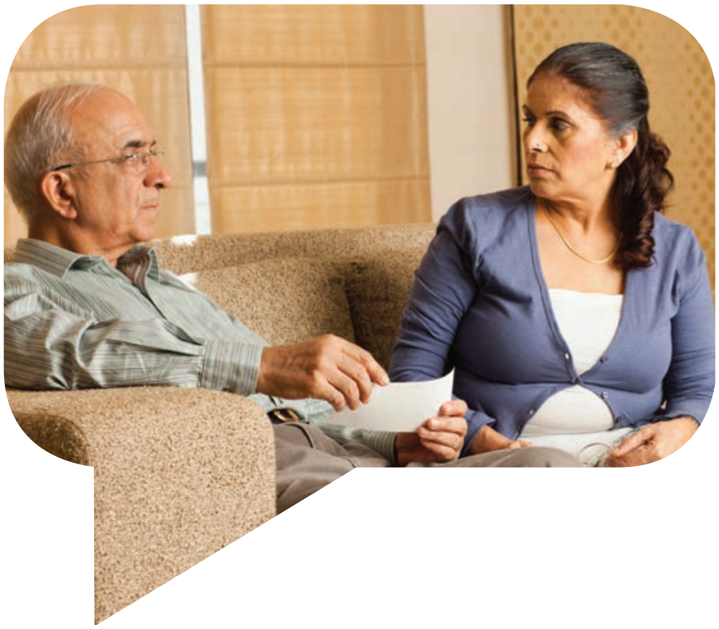

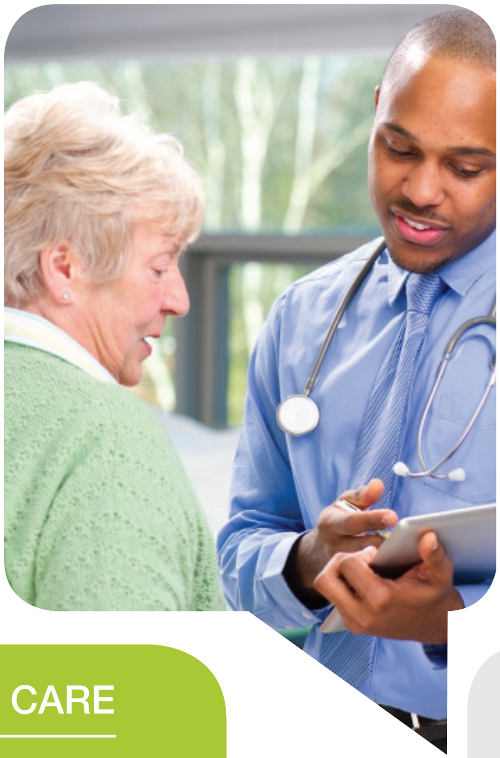

ADVANCE CARE PLANNING | GOALS OF CARE

CONVERSATIONS MATTER

A GUIDE FOR MAKING HEALTHCARE DECISIONS

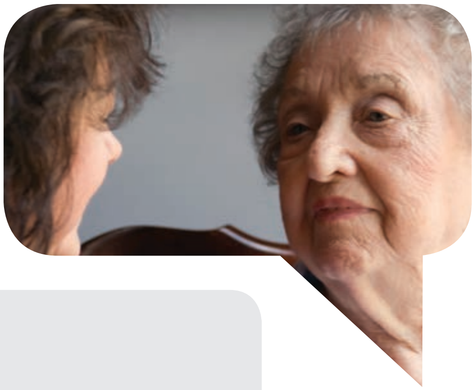

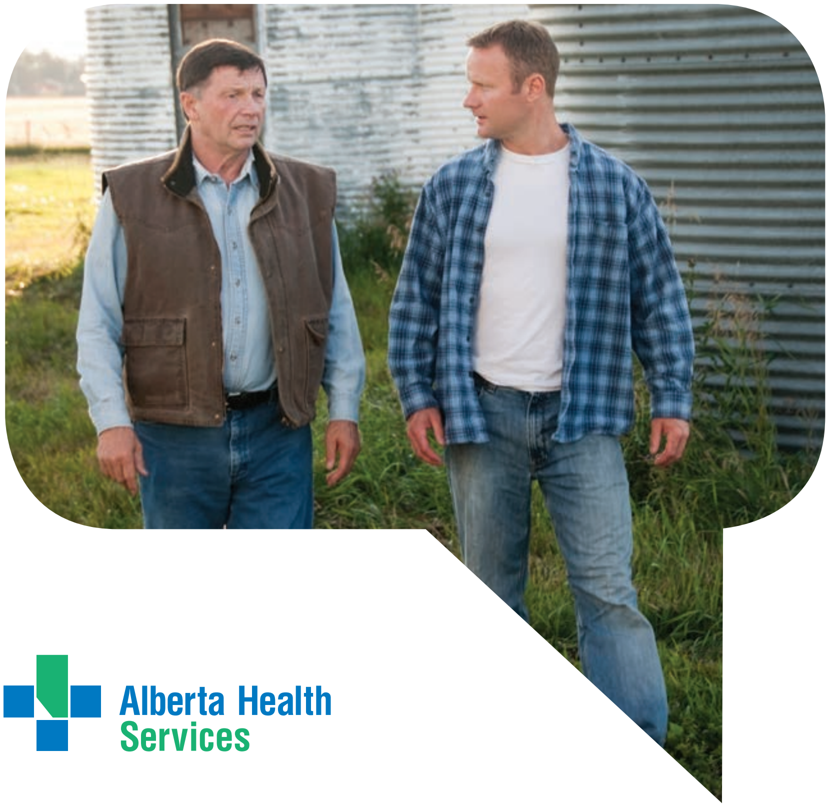

Alberta Health Services

What is Advance Care Planning?

Advance Care Planning is a way to help you think about, talk about and document your wishes for health care. It is a process that can assist you in making healthcare decisions now and for the future.

What are the benefits?

If there is a time when you are unable to speak for yourself, it is important that your loved ones and your healthcare team understand your wishes for health care.

None of us know what tomorrow might bring, or can predict our future health. Planning today can ensure that your wishes are known, no matter what the future holds.

Advance Care Planning may bring comfort and peace of mind to you, your family, and to those who may have to make healthcare decisions on your behalf.

> "I want my family to know my feelings about my future health care. That way, if they're faced with making decisions on my behalf they'll have peace of mind."

Who is it for?

Everyone. We can all benefit from Advance Care Planning. If an unexpected event or change in your health occurs and you are unable to make decisions about your health care, planning in advance ensures your wishes will be known.

When is a good time to start?

Now. It is important to begin Advance Care Planning conversations before you face a crisis or become seriously ill.

Imagine that without warning, you are seriously injured in a car crash. You are admitted to a hospital intensive care unit and are no longer able to communicate with anyone. Your heartbeat and breathing can only continue with artificial support. Your doctors believe it is unlikely you will recover.

Imagine your ability to make your own decisions is gone. You live at a care facility. You cannot feed yourself and no longer know who you are, who your family members are, or what happens from one moment to the next. You will never regain your ability to communicate meaningfully with others and your condition will become worse over time.

Imagine you have lived many years with an illness that is getting worse despite treatment and you are nearing the end of your life.

Will your family and healthcare team know your wishes?

How do I begin?

Think Learn Choose Communicate Document

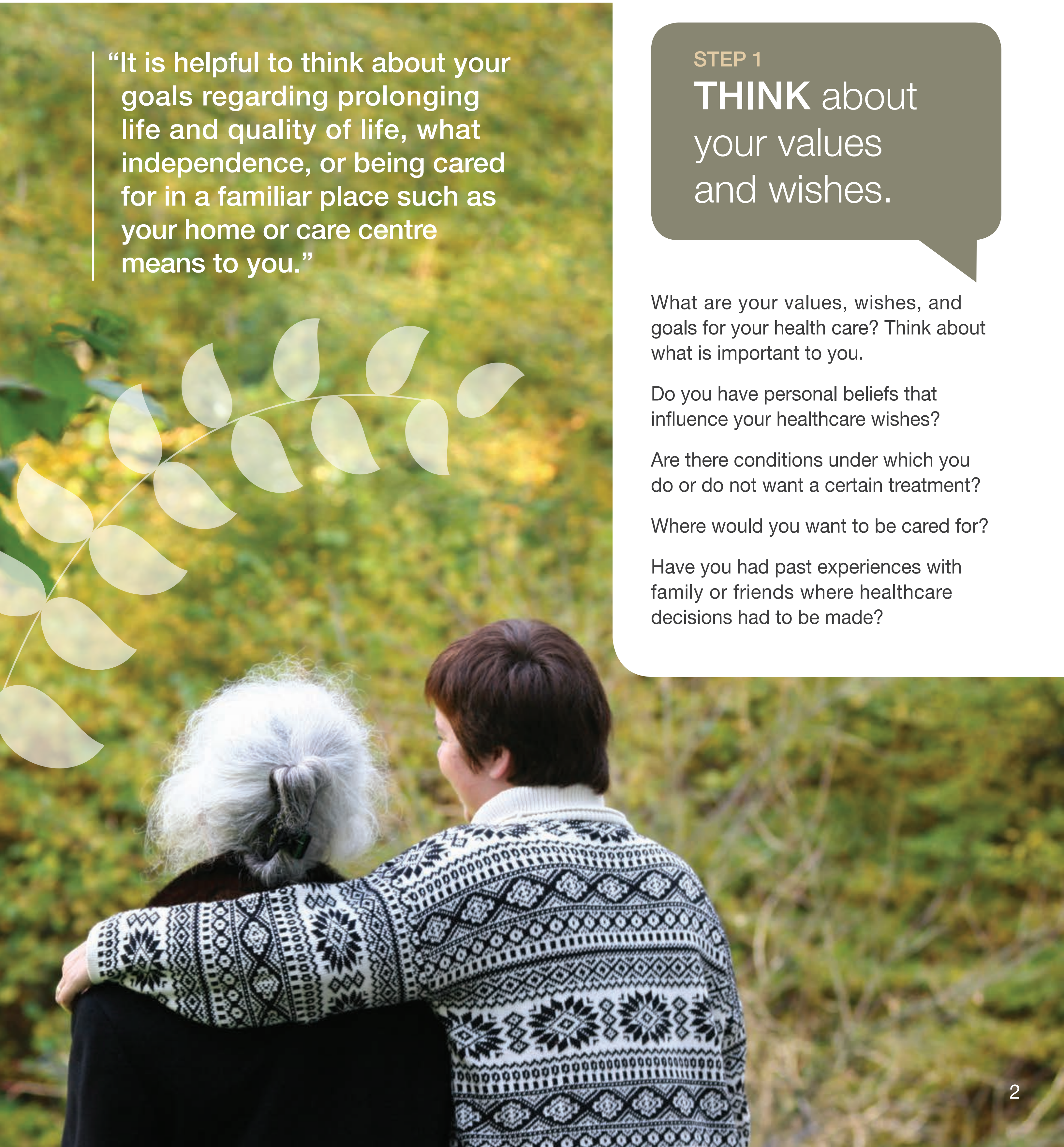

"It is helpful to think about your goals regarding prolonging life and quality of life, what independence, or being cared for in a familiar place such as your home or care centre means to you."

STEP 1

THINK about your values and wishes.

What are your values, wishes, and goals for your health care? Think about what is important to you.

Do you have personal beliefs that influence your healthcare wishes?

Are there conditions under which you do or do not want a certain treatment?

Where would you want to be cared for?

Have you had past experiences with family or friends where healthcare decisions had to be made?

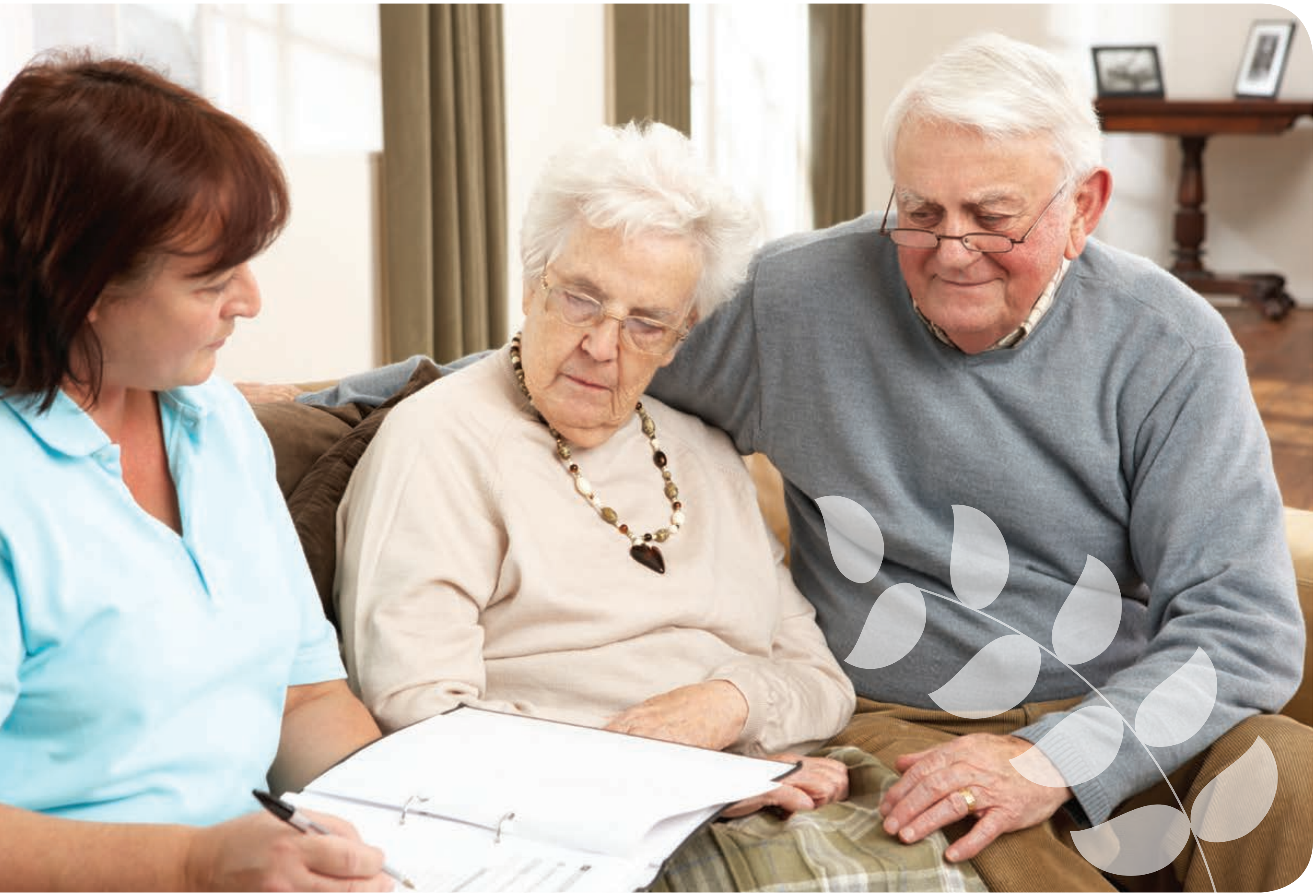

STEP 2

LEARN about your own health.

If you have an existing medical condition, it's important to talk to your doctor or other healthcare providers. You can ask about your prognosis, that is, what you might expect to experience in the future as a result of your medical condition. You can learn about the possible medical treatments for your condition and what to expect from these treatments. You can understand the types of healthcare decisions you may need to make.

> "While we can never predict exactly what life will bring, I can talk to you generally about what other patients with your condition have experienced."

STEP 3

CHOOSE someone to make decisions and speak on your behalf.

Unexpected or sudden medical events can leave us unable to communicate our wishes. Other medical conditions can slowly take away our ability to communicate or make decisions about our health care. This is why another important step in the Advance Care Planning process is to choose and legally appoint someone who can speak for you in the event you are unable to make medical decisions for yourself. This person would be your agent.

An agent can be anyone you choose, such as a family member or close friend. It is very important to discuss your values and wishes with the person you are considering to be your agent.

Some of the things you may want to consider:

Do I trust this person to make healthcare decisions with my healthcare team based on my values and wishes?

Are they able to communicate clearly?

Would they be able to make difficult decisions in stressful situations?

Is this person willing and available to speak for me if I were unable to make healthcare decisions for myself?

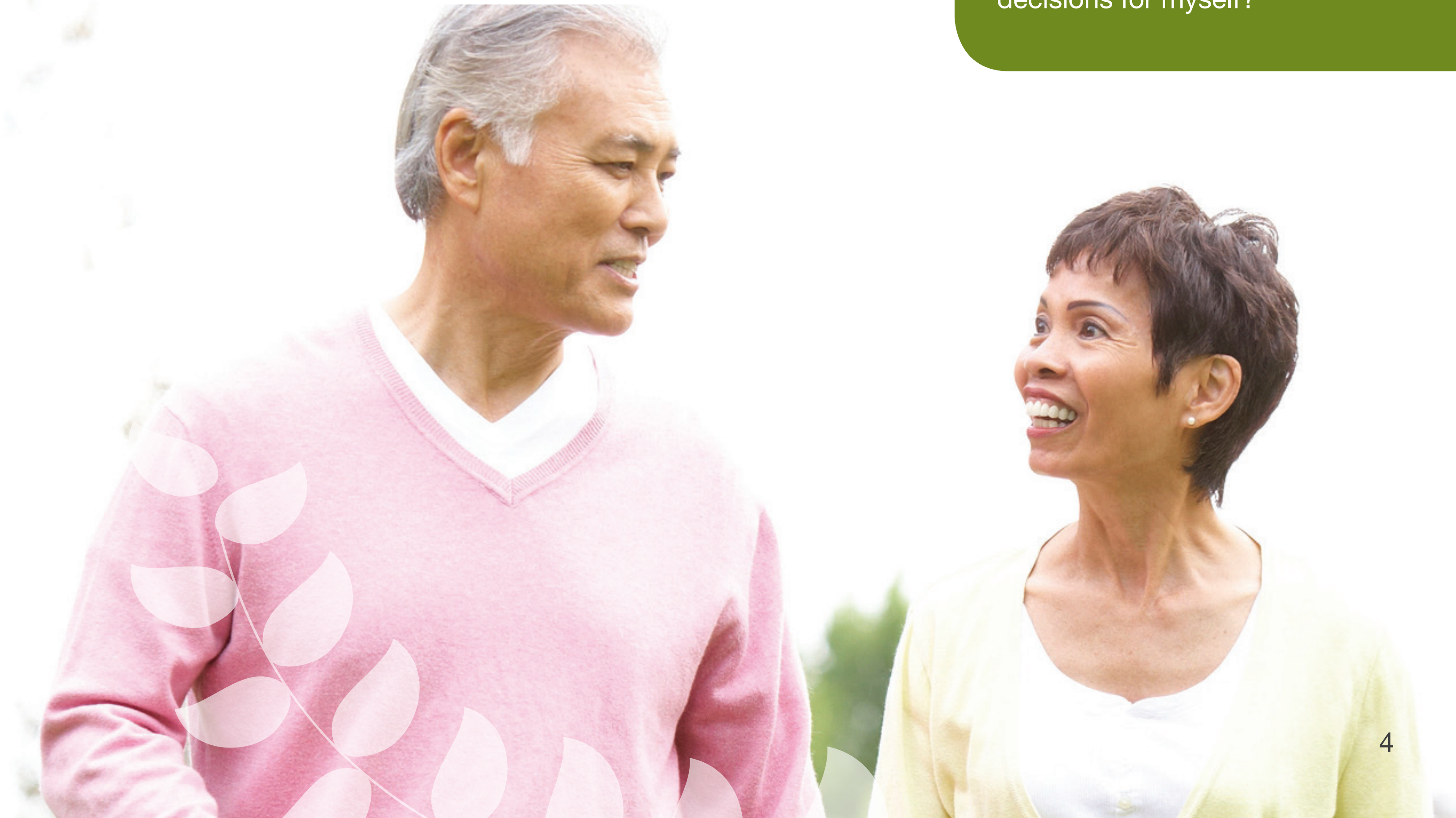

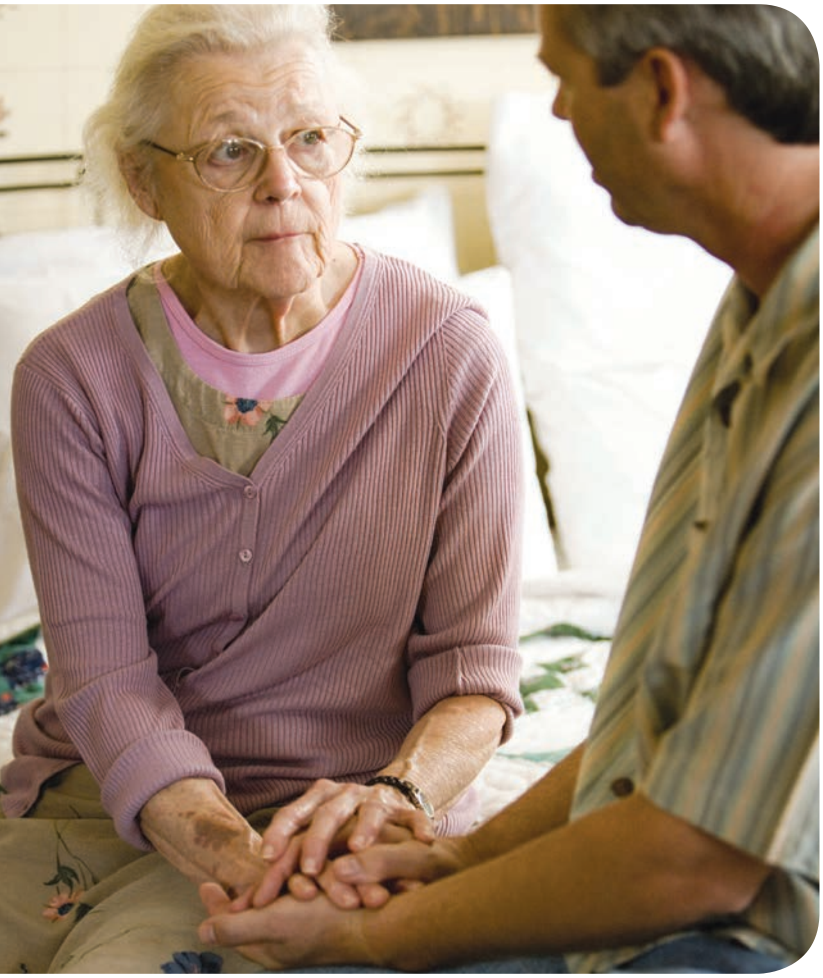

"I spoke with my son about my advance care plan. At first he wasn't keen. He tried to tell me I was needlessly worrying, and that I am fit and healthy. I know he gets upset thinking of a time when I won't be here.

I explained that I intend to stay well, but in case something happens and I can't speak for myself, I want him to be comfortable making medical decisions with my doctors. I was able to tell him what I value about my health and what my priorities are if I get very sick or when I'm dying. But, more importantly I know he won't feel burdened or worried about making the right decisions for me, because now he knows the kinds of things that I value in my health.

Later, I called the rest of my family. I wanted to make sure they knew my son was my agent and what we'd talked about. I don't want any misunderstandings when I'm sick."

STEP 4

COMMUNICATE your wishes and values about health care.

Likely the most important part of advance care planning is the conversations that you have over time with your designated agent, loved ones and healthcare team. Talking about your health with loved ones may be difficult, but understanding your wishes in advance will help them later on. It will help make an already stressful and uncertain time less difficult.

These conversations can also be helpful in determining the medical approach to your care, or Goals of Care Designation, that best reflects your wishes and health circumstances.

Your wishes and values may change over time or with changes in your health, so be sure to keep the conversation open and communicate these changes.

STEP 5

DOCUMENT in a Personal Directive.

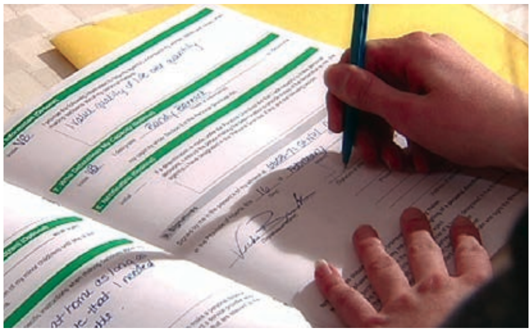

The next step in advance care planning is to document your plan. In Alberta, the legal document for this is called a Personal Directive. In a Personal Directive you choose an alternate decision maker to be your 'agent' – that is the person who can collaborate with your healthcare team if you are unable to do so. Your agent makes healthcare decisions on your behalf, in keeping with your wishes. You can also write down any other information about your wishes and values related to health care in your Personal Directive.

The Personal Directive only comes into effect if there is ever a time that you are unable to make decisions about your health care. It can be helpful in reducing conflict or distress and bringing comfort to those who are close to you, because it clearly states who your healthcare decision maker is and can provide guidance about your wishes.

Your Personal Directive can, and should be reviewed any time you have a change in your health circumstances or your wishes and values. If you would like more information about Personal Directives, you can contact the Office of the Public Guardian for assistance.

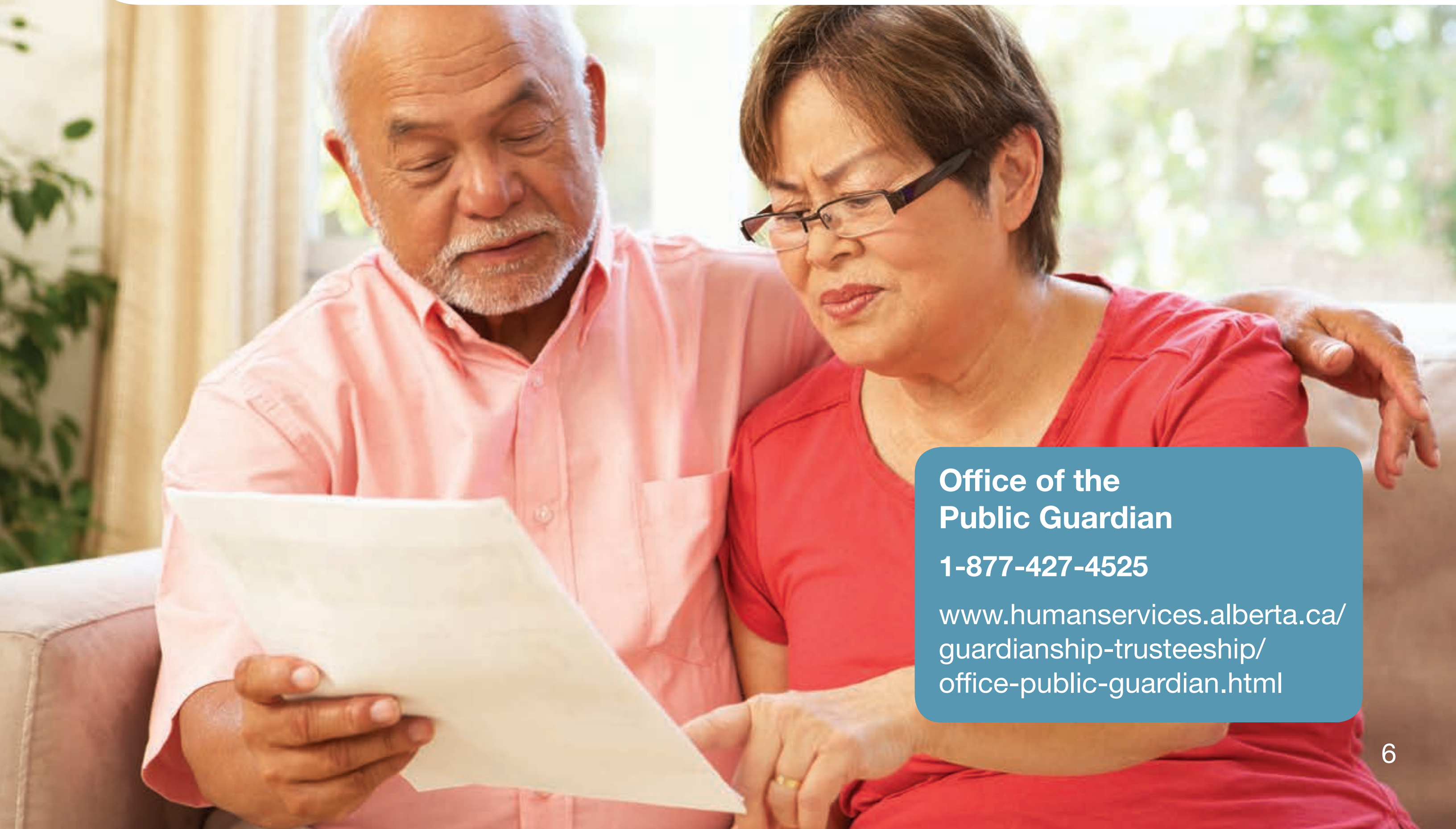

Office of the Public Guardian

1-877-427-4525

www.humanservices.alberta.ca/guardianship-trusteeship/office-public-guardian.html

Goals of Care Designations

Advance care planning conversations can help guide healthcare decisions. Goals of Care Designations are used by your healthcare providers to describe the general aims of your health care and the preferred location of that care. In a medical emergency, your Goals of Care Designation guides your healthcare team to provide timely care that best reflects your health condition, the treatments that will be of benefit to you, and your own wishes and values.

"When a patient talks to me about what is important to them, and what their hopes are in light of what they are experiencing with their health, we can work together to define the goals for their care. These conversations help me understand the patient's unique wishes in the context of their health circumstances so that an appropriate Goals of Care Designation can be determined."

There are three general approaches to care, or Goals of Care Designations:

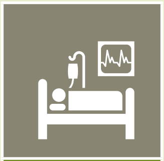

Resuscitative Care – The focus is to prolong or preserve life using any medical or surgical means including, if needed, resuscitation and admission to Intensive Care.

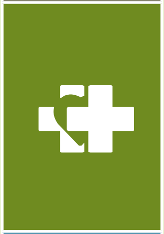

Medical Care – Medical tests and interventions are used to cure or manage an illness as well as possible but don't use resuscitative or life support measures. This is appropriate when resuscitative and life support measures won't work or when the person chooses not to receive such treatments. Medical care can be provided in many locations, depending on the person's wishes and values as well as medical appropriateness.

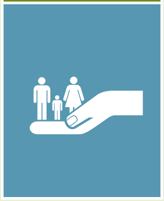

Comfort Care – In this approach to care, the aim of medical tests and interventions are for optimal symptom control and maintenance of function when cure or control of an underlying condition is no longer possible or desired. Transfer to a hospital may occur in order to better understand or control symptoms.

Within these three main approaches to care (Resuscitation, Medical, and Comfort Care), there are sub-categories. These are used to further define and communicate your goal of care designation to healthcare providers.

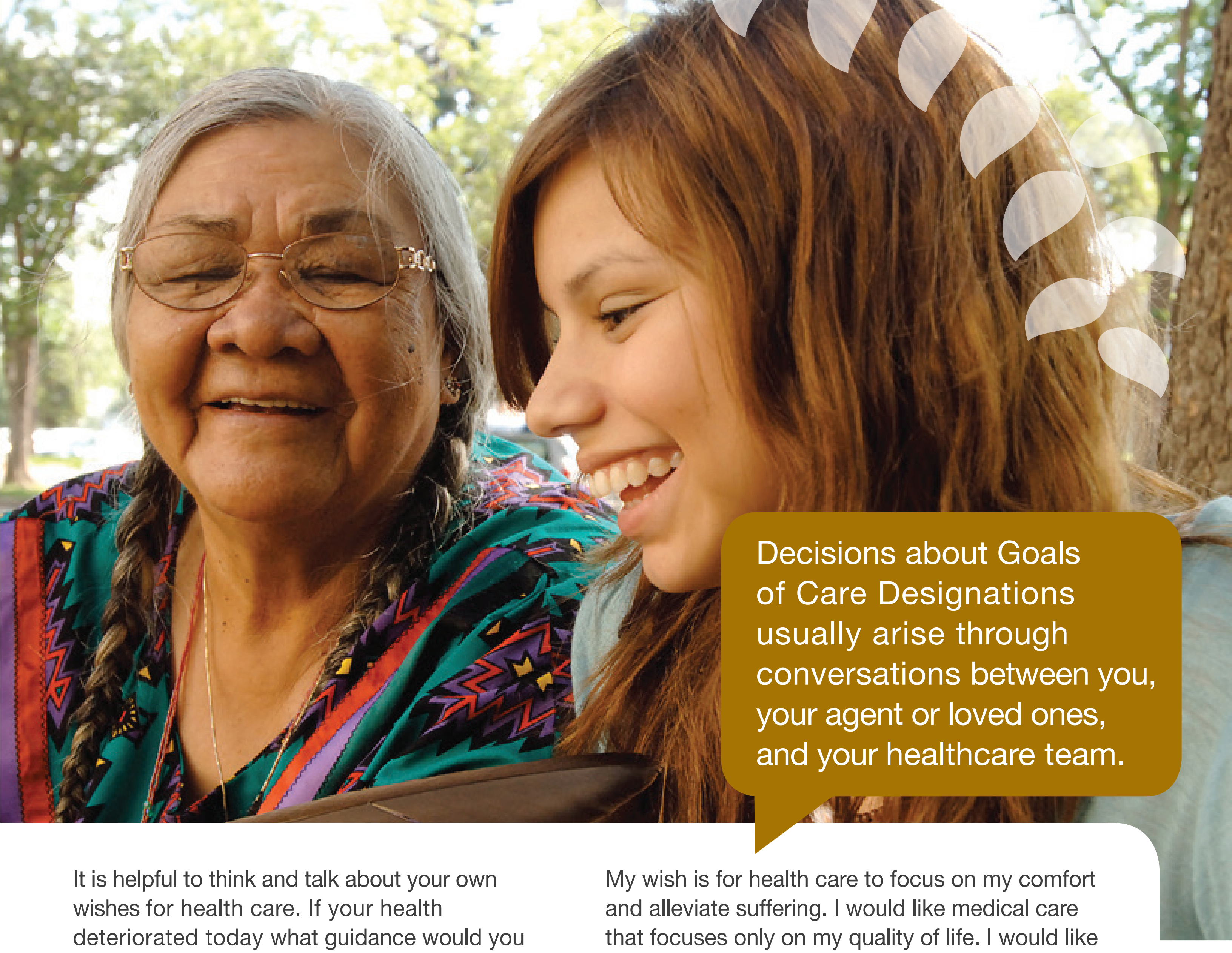

It is helpful to think and talk about your own wishes for health care. If your health deteriorated today what guidance would you give to your healthcare team and family?

Here are some starting points for your own conversations:

My wish is to use all medical therapies including resuscitation and artificial life-sustaining treatments in intensive care, to keep me alive if at all possible. (R)

Length of life is most important to me regardless of my recovery or changes in the quality of my life.

My wish is for full medical care but without the use of resuscitation or artificial life-sustaining intensive treatments, if these are either unlikely to prolong my life or restore me to a certain quality of life. (M)

My wish is for health care to focus on my comfort and alleviate suffering. I would like medical care that focuses only on my quality of life. I would like to avoid being kept alive by medical treatments, resuscitation or artificial life-sustaining intensive treatments. (C)

I am accepting of treatments that try to fix problems but if I'm not getting better or going to achieve a certain quality of life, I would want to switch to focusing only on my comfort and allowing natural death to occur.

It is important for me to discuss what I mean by quality of life.

Under what circumstances would I want to avoid being kept alive by medical treatments, resuscitation or intensive care?

What kind of changes to my health or life in the future might affect my wishes?

When are Goals of Care Designations Discussed or Changed?

Your healthcare team will approach you to discuss the goals for your care:

- When you are admitted to a hospital or care facility
- Before you have surgery
- When you are transferred from one healthcare facility to another
- If you come to an emergency department or urgent care centre
- At your annual check up with your family doctor
- Any time there is a change in your health circumstances
- At your discretion, whenever you are preparing or reviewing your advance care plan

In most cases, you, your agent, your loved ones and healthcare team will agree about the Goals of Care Designation that is most appropriate for you. However, if there is a time when reaching an agreement is hard to do, there is a Dispute Resolution process in place to help support decision-making. Ask your healthcare provider for more information.

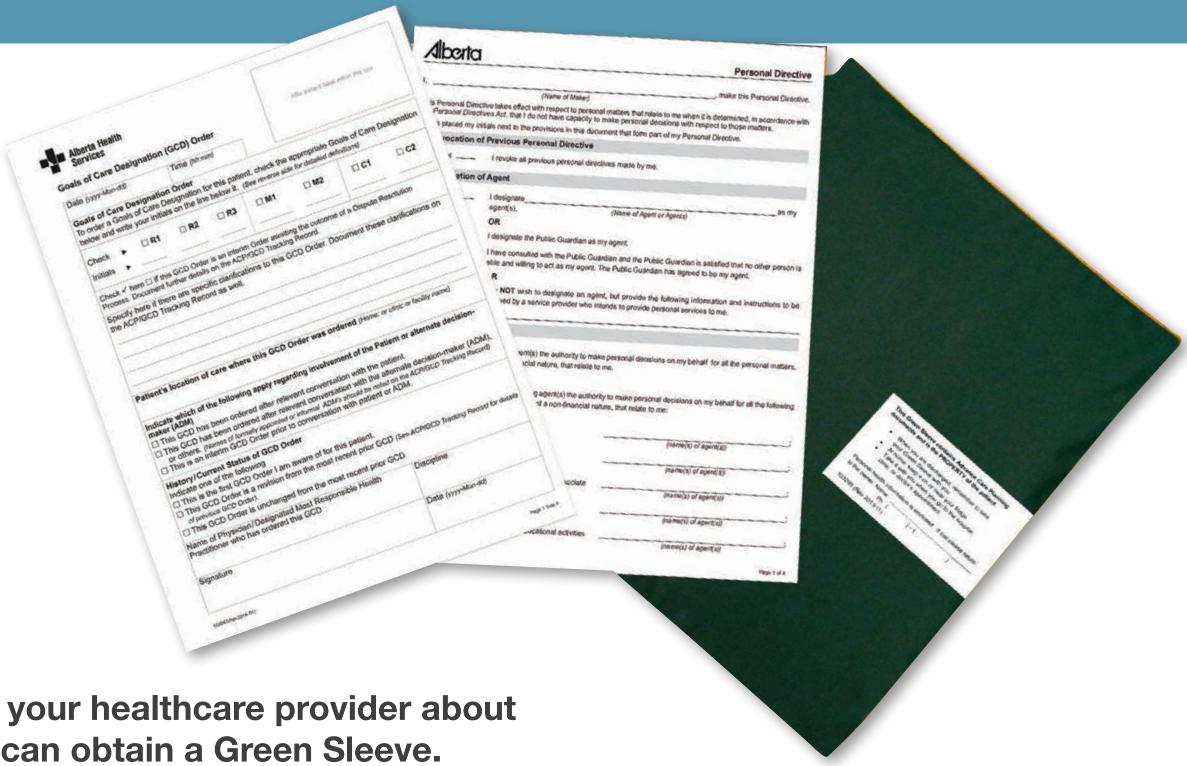

Speak to your healthcare provider about how you can obtain a Green Sleeve.

The Goals of Care Designation order is documented on an Alberta Health Services form by your physician or nurse practitioner and is recognized across healthcare services. This order, along with a copy of your Personal Directive and any other advance care planning documentation is kept in a plastic Green Sleeve. When you are at home, you should keep your Green Sleeve on or near your fridge as that is where healthcare providers such as Emergency Medical Services will look for it upon entering your home. Any time you go to the hospital or to any healthcare provider, take your Green Sleeve with you and be sure it comes with you when you leave. Your Green Sleeve is an important part of communicating your advance care planning and Goals of Care Designation.

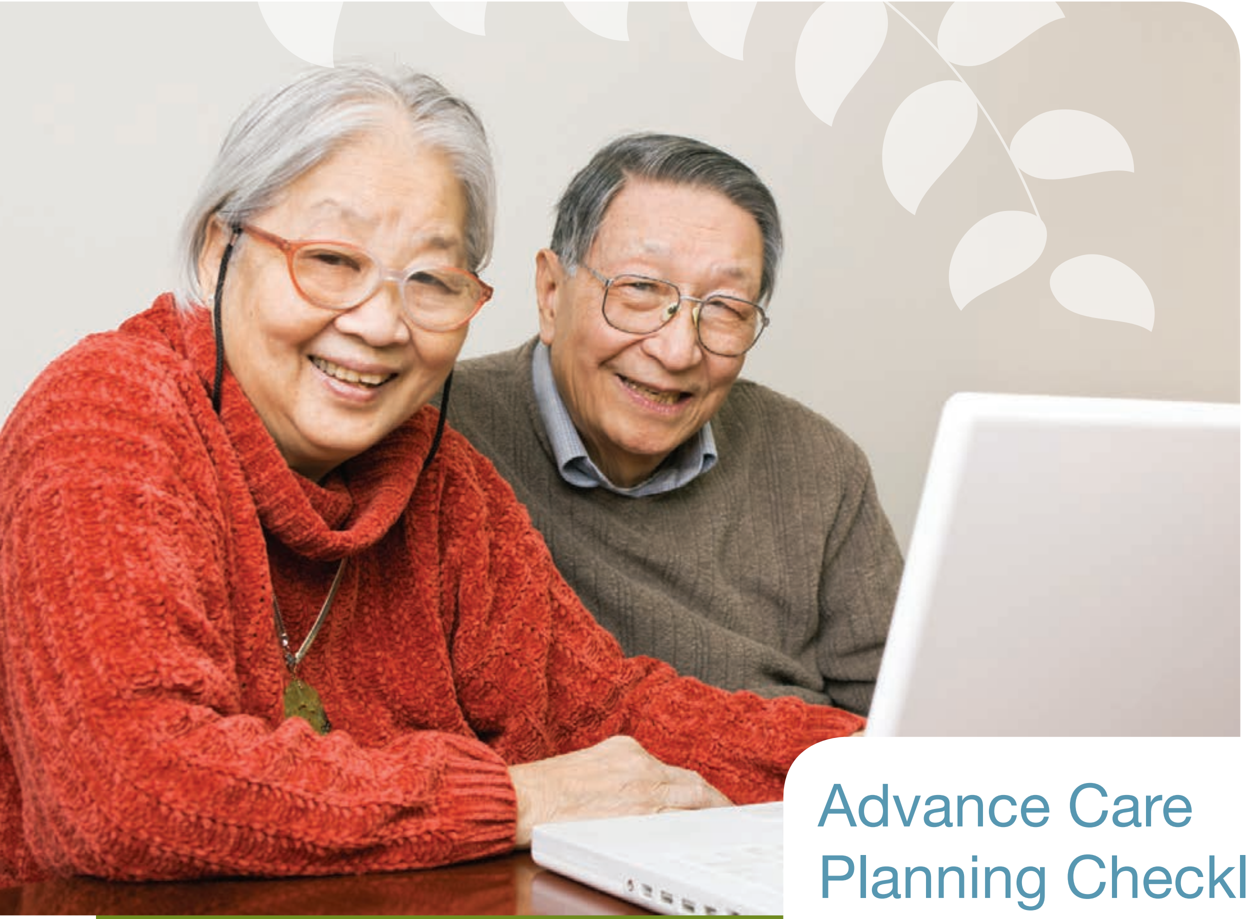

Advance Care Planning Checklist

- ❑ I have thought about my values and beliefs and personal goals as they relate to my health care
- ❑ I have spoken to my healthcare provider(s) about my current heath and what future healthcare decisions I might need to make
- ❑ I have chosen an agent to whom I have spoken to about my wishes
- ❑ I have discussed my healthcare treatment wishes with my family and/or trusted friends
- ❑ My doctors and I have spoken together about my Goals of Care Designation
- ❑ I have completed a Personal Directive and given copies to my agent and healthcare provider(s)
- ❑ I have a Green Sleeve to hold my documents (including my Personal Directive and Goals of Care Designation order) and have placed it on or near my fridge
- ❑ I will bring my Green Sleeve to the hospital or other healthcare appointments whenever I go

If you would like to obtain a wallet card identifying contact information for your agent, in the event of a medical emergency, contact the Office of the Public Guardian:

1-877-427-4525

Resources for healthcare decision making:

- Brochures
- Videos

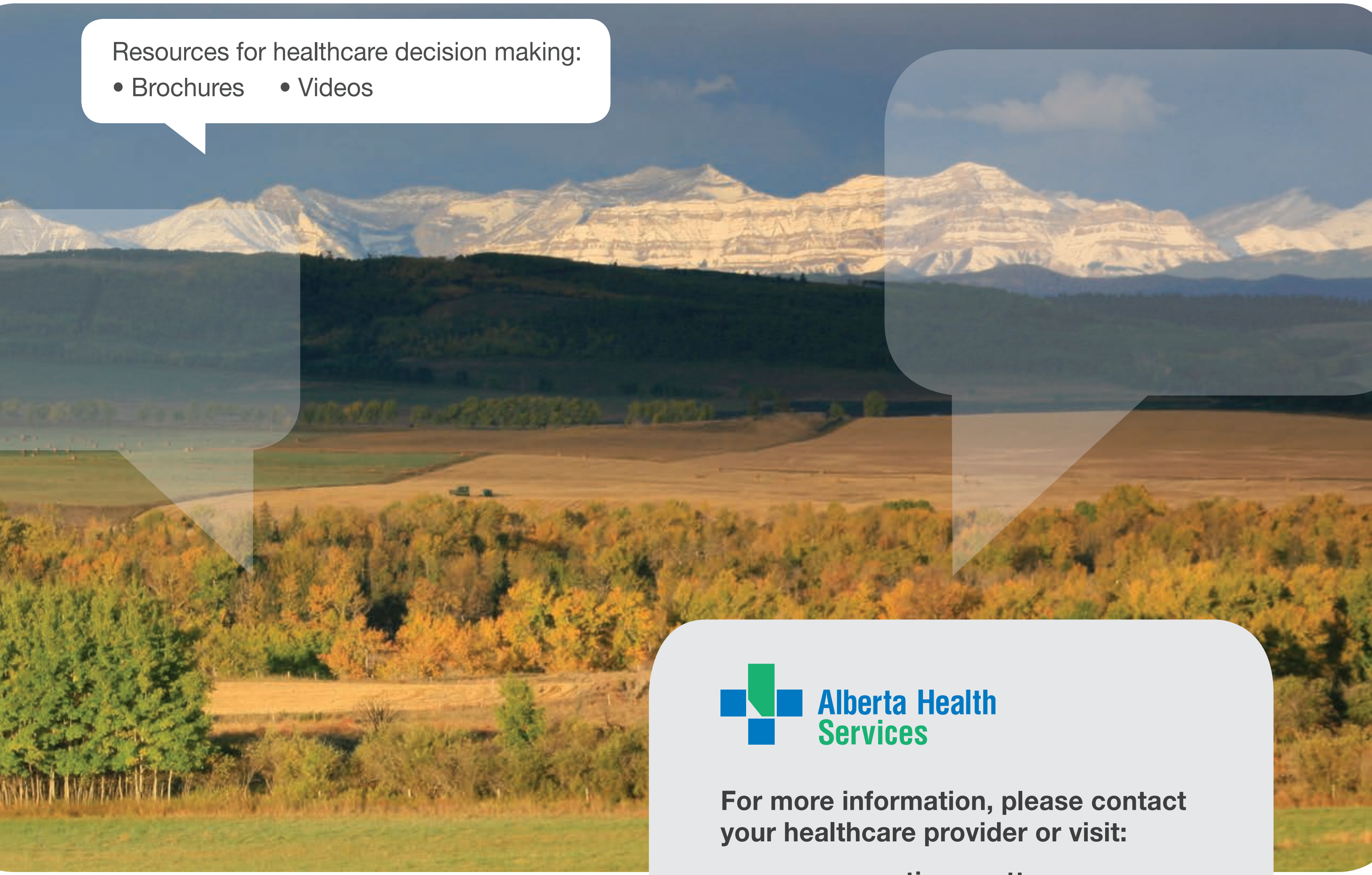

Alberta Health Services

For more information, please contact your healthcare provider or visit:

www.conversationsmatter.ca

104289 (2014-01)
